# Collectanea Medico-Chirurgica; or, Cullings from the Case-Book of a General Practitioner in Medicine, Surgery, and Midwifery

**Published:** 1846-07

**Authors:** 


Art. III.—*Collectanea Medico-Chirurgica; or, Cullings from the Case-Book of a General Practitioner in Medicine, Surgery, and Midwifery.* By W. Maclure, Esq., Surgeon, &c. &c. &c.—*London,* 1845. 8vo, pp. 192.

This collection of cases, the author in his preface informs us, is given to the public with a view "in some degree to exhibit the nature and extent of the labours of the general practitioner," and to show the injustice, as Mr. Maclure believes, "of the recent attempts of the Council of the College of Surgeons of England to degrade him" [the general practitioner] "below his recent level." The author makes the singular announcement, that in his miscellaneous volumes, he does not consider it necessary to "load his pages and swell the size of his book" with the "uninteresting details" of ordinary practice, "*such as*" the protean forms of gastric and hepatic derangements, of gout and rheumatism, of the operation for hernia, stone, tumours, &c.; yet these and the other diseases enumerated by Mr. Maclure (p. vi) are at once the most important and the most common, and in regard to which no man would consider "details," if judiciously selected and stated, as "uninteresting" or unneeded.

The volume is multifarious in its nature. One or two of the subjects discussed have little bearing on practical medicine, and others have nothing very unusual to recommend them. There is one case, however, extremely creditable to Mr. Maclure's surgical management. It is headed "Partial obliteration of the urethra cured by the caustic potash;" and although it has been already elsewhere published, a slight recapitulation of some points in the treatment may here be given.

The patient when he came under the author's care, had been for sixteen years a sufferer from a fistulous aperture in the perineum, through which his urine was discharged instead of by the urethra. The disease was originally caused by chancrous sores which spread downwards from the groin. The orifice of the fistula, with its glistening lining membrane, was situated rather less than half the distance between the scrotum and anus. A probe could be introduced by the fistula backwards into the bladder, but not forward to the glans penis, being stopped by a cul-de-sac, in which the anterior portion of the canal terminated. The obstruction was situated five or six inches from the orifice of the urethra, and *seemed to occupy an inch and a half of the canal.* The treatment consisted in the application of an armed bougie, which was first introduced on the 15th of November, 1822. On the 17th of January, 1823, a catheter could be passed into the bladder. On the 20th, the urine was withdrawn by means of a gum-catheter, introduced along the urethra, instead of flowing by the fistula. On the 26th of February, the patient regained the natural use of the canal.

There are several other papers of some interest, though few of them are sufficiently so to justify their being resuscitated from the pages of the journals in which they originally appeared.

